# Nanoscale Extracellular Vesicle-Enabled Liquid Biopsy: Advances and Challenges for Lung Cancer Detection

**DOI:** 10.3390/mi15101181

**Published:** 2024-09-24

**Authors:** Adeel Khan, Faisal Raza, Nongyue He

**Affiliations:** 1 State Key Laboratory of Bioelectronics, School of Biological Science and Medical Engineering, National Demonstration Center for Experimental Biomedical Engineering Education, Southeast University, Nanjing 210096, China; 2 School of Pharmacy, Shanghai Jiao Tong University, Shanghai 200240, China; faisalraza@sjtu.edu.cn

**Keywords:** lung cancer, liquid biopsy, extracellular vesicles, exosomes, non-invasive diagnosis, biomarkers, circulating tumor cells, circulating tumor DNA

## Abstract

Lung cancer is responsible for the death of over a million people worldwide every year. With its high mortality rate and exponentially growing number of new cases, lung cancer is a major threat to public health. The high mortality and poor survival rates of lung cancer patients can be attributed to its stealth progression and late diagnosis. For a long time, intrusive tissue biopsy has been considered the gold standard for lung cancer diagnosis and subtyping; however, the intrinsic limitations of tissue biopsy cannot be overlooked. In addition to being invasive and costly, it also suffers from limitations in sensitivity and specificity, is not suitable for repeated sampling, provides restricted information about the tumor and its molecular landscape, and is inaccessible in several cases. To cope with this, advancements in diagnostic technologies, such as liquid biopsy, have shown great prospects. Liquid biopsy is an innovative non-invasive approach in which cancer-related components called biomarkers are detected in body fluids, such as blood, urine, saliva and others. It offers a less invasive alternative with the potential for applications such as routine screening, predicting treatment outcomes, evaluating treatment effectiveness, detecting residual disease, or disease recurrence. A large number of research articles have indicated extracellular vesicles (EVs) as ideal biomarkers for liquid biopsy. EVs are a heterogeneous collection of membranous nanoparticles with diverse sizes, contents, and surface markers. EVs play a critical role in pathophysiological states and have gained prominence as diagnostic and prognostic biomarkers for multiple diseases, including lung cancer. In this review, we provide a detailed overview of the potential of EV-based liquid biopsy for lung cancer. Moreover, it highlights the strengths and weaknesses of various contemporary techniques for EV isolation and analysis in addition to the challenges that need to be addressed to ensure the widespread clinical application of EV-based liquid biopsies for lung cancer. In summary, EV-based liquid biopsies present interesting opportunities for the development of novel diagnostic and prognostic platforms for lung cancer, one of the most abundant cancers responsible for millions of cancer-related deaths worldwide.

## 1. Introduction

Lung cancer is the most widespread cancer with the worst chance of survival [1]. Its onset is primarily linked to smoking and exposure to certain environmental factors [2]. The most common categories of lung cancer are non-small cell lung cancer (NSCLC) and small-cell lung cancer (SCLC) [3]. Based on the number of cases, NSCLC was more prevalent (85%) than SCLC (15%). NSCLC is further classified into three subcategories: squamous cell carcinoma (SCC), adenocarcinoma, and large-cell carcinoma. Among the subtypes, prevalence is led by adenocarcinoma with 40% of cases, followed by SCC with 25% of cases, and large cell carcinoma with 10% of cases. SCC is strongly associated with smoking. Although large cell carcinoma is the least common subtype, it can spread quite quickly in any part of the lung in the form of poorly differentiated cells exhibiting a high growth rate [4].

The global burden of lung cancer is substantial [5]. The International Agency for Research on Cancer (IARC) platform revealed 1.8 million deaths and 2.4 million new incidences of lung cancer in 2022, showing the re-emergence of lung cancer as the most abundant cancer in terms of new cases and cancer-related deaths worldwide [6]. Projections for China indicate that by 2040, lung cancer cases will reach 6.85 million, with 5.07 million fatalities attributed to this disease [7]. These data emphasize the seriousness of the threat of lung cancer to public health and call for effective prevention and management measures [8,9]. Lung cancer presents with a wide range of symptoms, and patients are often asymptomatic in the early stages of the disease. Although symptoms may be present, they are typically nonspecific and resemble common benign conditions. A persistent cough that lasts for more than three weeks, especially if accompanied by blood or mucus, as well as recurring lung diseases such as bronchitis or pneumonia, persistent chest pain, and weight loss, may also be indicative of lung cancer. However, these symptoms can take years to develop or may only appear at advanced stages of the disease [10].

The worst survival ratio of lung cancer can be attributed to its stealth progression to advanced stages without detection, as conventional detection approaches, such as tissue biopsy and imaging modalities, are incapable of ensuring its early detection. Therefore, it is crucial to search for new strategies to enable its timely diagnosis. The present gold standard approach for the detection of lung cancer is tissue biopsy [11]; however, it is highly invasive and not suitable for specific purposes, such as routine screening, predicting or evaluating the effectiveness of treatment, monitoring the presence of remaining illness, and promptly detecting relapse. Additionally, because of the small sample size, it does not provide information about tumor heterogeneity [12]. These techniques have an associated risk of complications such as infections and bleeding. In conclusion, while lung cancer diagnosis traditionally relies on tissue biopsy, its inherent limitations, such as intrusive operation, high cost, risk causation of infection, bleeding, anxiety in patients, and insufficient information because of tumor heterogeneity, cannot be overlooked. These limitations highlight the inadequacy of tissue biopsy techniques and call for novel techniques that can address these issues [13]. Advancements aimed at developing and improving non-invasive techniques for lung cancer detection can be highly advantageous for enhancing patient care by providing safer, reliable, and repeatable approaches for diagnosing and monitoring lung cancer [14].

Likewise, imaging techniques are extensively used as non-invasive approaches for the detection of lung cancer, with low-dose computed tomography (LDCT) being a prominent method [15]. Through these scans, radiologists can identify the lung nodules. Lung nodules are considered serious as they can be early indicators of lung cancer in most cases. Individuals with high-risk lung nodules are referred for surgery to remove suspicious lung nodules [16]. Additionally, non-invasive imaging tests face challenges, such as a lack of skilled personnel to analyze the results in low-income countries or remote areas and high false-positive rates, particularly in regions with prevalent fungal lung infections [17]. Techniques that limit the deficiencies associated with imaging modalities, specifically LDCT, such as laborious analysis of CT scans to detect lung nodules, reduced false-positive rates, or differentiation between malignant and benign lung nodules from CT scans, would greatly improve the avenue of lung cancer detection via non-invasive imaging [18].

**Figure 1:**
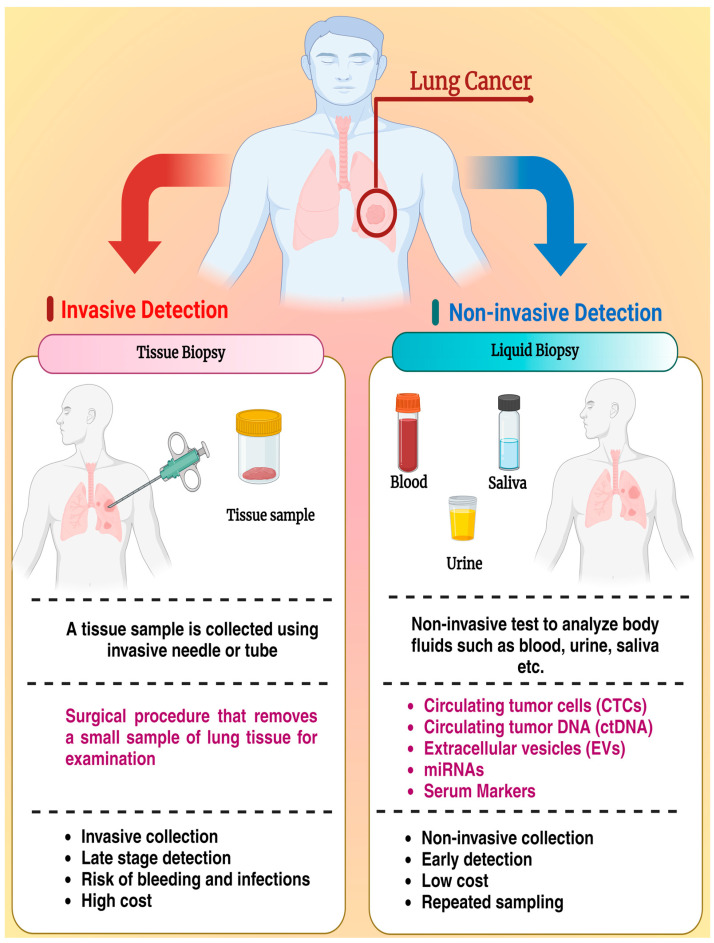
Lung cancer diagnosis and the two contrasting approaches: conventional tissue biopsy vs. liquid biopsy. As shown in purple text the target of tissue biopsy is to get a small sample of lung tumor for examination, while liquid biopsy targets circulating tumor cells (CTCs), circulating tumor DNA (ctDNA), extracellular vesicles (EVs) and microRNA (miRNA) and serum markers to offer a non-invasive and dynamic alternative.

There is a huge demand for innovative tools that are non-invasive and facilitate the precise detection of lung cancer at earlier stages. Modern research indicates that liquid biopsy techniques are comprehensive and dynamic tools that can be used for cancer detection and monitoring. Liquid biopsy is non-invasive by nature, focusing on body fluids for the detection of biomarkers such as circulating tumor cells, circulating tumor DNA, extracellular vesicles, and others, and offers advantages such as suitability for early detection, real-time monitoring of cancer progression and response to treatment, low cost, and amenable to multiple resampling, making it a superior method to conventional tissue biopsy, as shown in Figure 1 [19].

## 2. Liquid Biopsy Approach

In liquid biopsy, body fluid samples, such as blood, urine, saliva, pleural effusion, sweat, tears, and even bile, are collected to evaluate biomarkers that can offer crucial insights for cancer diagnosis and treatment [20]. Significant effort has been invested in discovering cancer-specific biomarkers in readily available biological samples. These biomarkers should be detectable with high accuracy and suitable for routine preventive screening. Optimal biomarkers should signal the presence of an asymptomatic condition and offer insights into the characteristics of the tumor, including its progression stage and responsiveness to treatment, among other important factors. To be considered effective, these biomarkers must be undetectable in healthy individuals without tumor cells, while being present at substantially elevated levels when tumor cells exist within the body [21].

**Figure 2:**
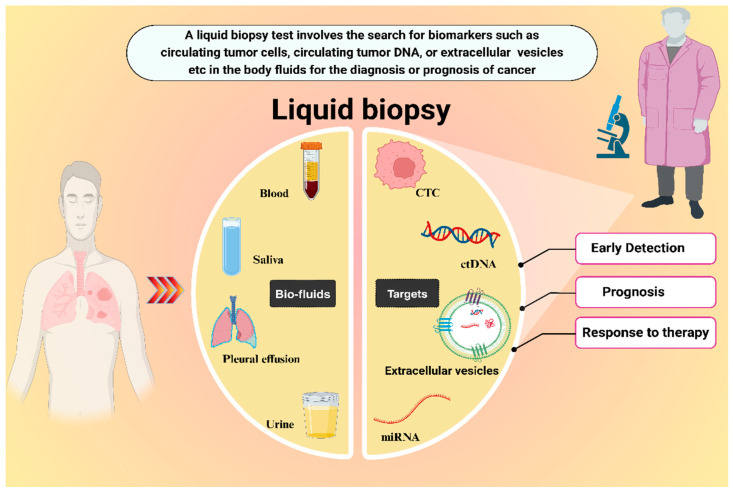
Schematic representation of the liquid biopsy approach for lung cancer detection.

Liquid biopsy primarily relies on biomarkers, such as circulating tumor cells (CTCs), circulating tumor DNA (ctDNA), extracellular vesicles (EVs), microRNAs (miRNAs), etc., as shown in Figure 2 [20].

Liquid biopsy has shown prospects for outperforming conventional methods in detecting lung cancer at an early stage. It can also provide information about the molecular landscape of the tumor by analyzing biomarkers in body fluids [22]. It has been anticipated that liquid biopsy will not only offer increased diagnostic accuracy but also be helpful in avoiding unnecessary invasive biopsies [13,23,24]. This approach offers several advantages, such as early detection, increased accessibility, and non-invasiveness, which in turn allow for repeated sampling to facilitate monitoring of treatment response and detection of cancer recurrence [25]. Therefore, unlike conventional methods involving tissue extraction, liquid biopsy can be a safer, more accurate, and repeatable alternative [14,26].

Biomarkers, such as CTCs, ctDNA, and miRNA, have been excessively hailed for their role as diagnostic markers; however, there are still limitations [27]. For instance, CTCs offer advantages like they can capture tumor heterogeneity, but are so rare during early stages, therefore, they are difficult to isolate. ctDNAs are fragments of tumor DNA that can be valuable indicators of genetic alterations but factors such as rarity during the early stages of the disease hinder their efficacy for detecting associated mutations. miRNAs are small non-coding RNAs with a stable presence in biofluids and can reflect tumor states, but they have issues with specificity and are condition dependent [22]. However, EVs and their versatile content represent a new paradigm as biomarkers for the liquid biopsy of lung cancer, facilitating its early diagnosis and progression monitoring [28].

## 3. Extracellular Vesicles (EVs)

**Figure 3:**
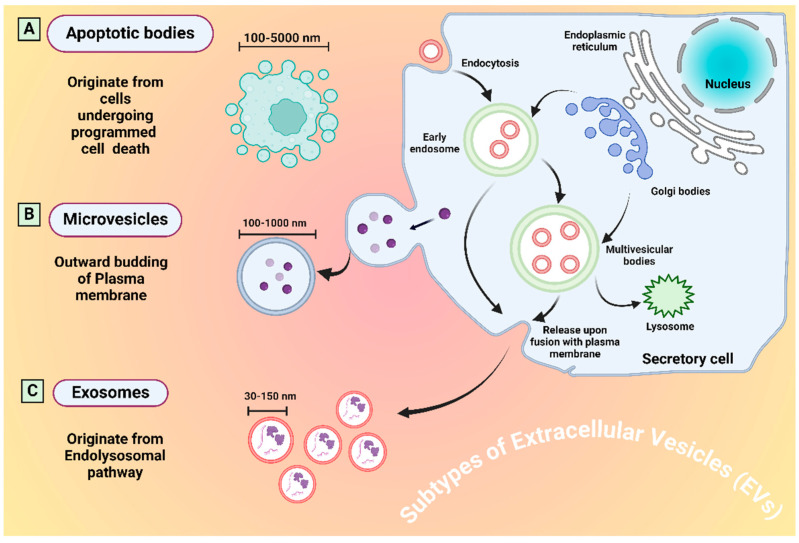
Biogenesis of the main subtypes (exosomes, microvesicles, and apoptotic bodies) of extracellular vesicles.

EVs can be defined as a heterogeneous collection of cell-derived particles with a lipid bilayer that lacks the ability to replicate. Subtyping of EVs is occasionally based on their size, density, molecular composition, or cellular origin. EVs can be categorized according to their size: those measuring under 200 nm are classified as small EVs, while those exceeding 200 nm are designated as large EVs. Based on the presumed biogenesis pathways, EVs released from cell internal compartments via multicellular bodies are called exosomes, whereas ectosomes are EVs released from the cell surface and can also be called microvesicles or microparticles. The main subtypes of EVs are microvesicles, exosomes, and apoptotic bodies (Figure 3).

Recent research has identified additional subtypes, such as large oncosomes, migrasomes, ectosomes, exomeres, supermeres, and membrane particles [29]. Information about the different subtypes of EVs, along with their sizes, origin, and identification markers are tabulated in Table 1. MISEV 2023 recommends the umbrella term EVs if the subtype is not confirmed [30]. The EVs field is rapidly advancing, resulting in a broadening of our understanding of EVs biology. EVs harbor diverse molecules such as proteins, nucleic acids (RNA and DNA), lipids and metabolites that are actively involved in intercellular communication. Cells consistently release EVs into the extracellular environment for exchanging biological information to influence pathological and physiological states. Their involvement has been revealed in all major diseases, including lung cancer. The role of EVs as communication tools between tumor cells has also been established. Cancer-derived EVs also contain cargo such as proteins, metabolites, mRNA, DNA fragments, and non-coding RNA such as miRNAs and lipids. As EVs facilitate the exchange of molecular cargo between donor and recipient cells, and hence are regarded as an integral component of the intercellular communication network [28].

Recent research has confirmed that EVs can be innovative biomarkers for liquid biopsy owing to their copious prevalence in various bodily fluids and for their implication in a range of physiological and pathological processes. Research has also confirmed that EVs can be isolated and utilized for clinical assessment, even during the early stages of a disease [38]. EVs contain various protein elements, including those found on the surface and within cells, which are significant in lung cancer progression and play a vital role in early identification and outcome prediction of the disease. Several exosomal membrane proteins, such as CD91, CD317, CD151, and CD171, have been identified as effective diagnostic biomarkers for lung cancer [39]. Furthermore, the surface proteins of exosomal membranes, such as EGFR, placental alkaline phosphatase, epithelial cell adhesion molecule (EpCAM), and Alix, serve as noteworthy indicators of long-term survival in patients with lung cancer [26].

## 4. Current State of EV-Based Liquid Biopsy for Lung Cancer

A large portion of research has reported the stable and abundant distribution of EVs in body fluids. The surface and luminal content of EVs have shown a strong association with the disease state, promising that EVs can be ideal candidates for clinical applications as biomarkers for disease diagnosis and prognosis [40]. In the context of lung cancer that kills over a million people every year, research efforts for early detection using EVs for liquid biopsy are a subject of great interest [41]. EVs as liquid biopsy markers allow the direct detection of cancer proteins from body fluids, and calnexin expression on the exosomal surface has been shown to be indicative of lung cancer [42]. EV-based biomarkers are preferred because they offer higher specificity, sensitivity, and enhanced reliability when compared to detecting biomarkers directly in traditional samples such as plasma, serum, and urine [43]. A substantial number of research articles have indicated EVs or EV cargo as sources of biomarkers for lung cancer liquid biopsies are tabulated in Table 2.

A crucial biomarker for detecting lung cancer is exosomal programmed death ligand 1 (PD-L1). The expression of PD-L1 in exosomes originating from lung cancer cells allows for the distinction between individuals with cancer and those who are healthy, as well as assists in determining the stage of the tumor. Notably, both the dual-labeled electrochemical method [58] and the integrated magneto-fluorescent exosome (iMFEX) sensor [59] exhibit high sensitivity and specificity for detecting PD-L1 positive exosomes, which is crucial for the accurate identification of lung cancer. The EV-anchor method was developed to detect PD-L1 positive EVs, and significant differences were observed between healthy individuals and patients with lung cancer [60]. CD63, a universal marker of exosomes in combination with PD-L1, is known to cause a worse response to immunotherapy, and when applied to clinical specimens, it has demonstrated exceptional capabilities in diagnosing and staging lung cancer [61]. Additionally, certain proteins found on exosome membranes, such as EGFR, placental alkaline phosphatase, EpCam, and Alix, have shown promise as potential prognostic indicators for lung cancer. Research has linked higher concentrations of these molecules on exosomal surfaces to decreased overall survival rates, indicating their potential value in forecasting long-term patient outcomes [62]. 

Current studies indicate that the search for biomarkers is increasingly focused on the contents of extracellular vesicles (EVs), particularly miRNAs and lncRNAs, for detecting lung cancer at an early stage. Current research indicates that exosomal miRNAs circulating in the body could be valuable biomarkers for detecting lung cancer. These small molecules, which regulate gene expression, have demonstrated potential in reducing false-positive rates when used alongside other diagnostic methods, such as low-dose CT scans. Furthermore, exosomal miRNAs are gaining recognition as standalone predictors of disease [63]. Despite the large quantities of circulating RNAses, miRNAs can be detected in serum, plasma, and sputum using quantitative reverse-transcription polymerase chain reaction [64]. Four miRNAs, namely miR-378a, miR-379, miR-139-5p, and miR-200b-5p, were identified in the plasma exosomes of 30 individuals to distinguish between patients with lung cancer and healthy individuals [65]. The utility of exosomal miRNAs in lung cancer diagnosis is promising. Dysregulated exosomal miR-486-5p and miR-451a have been shown to have diagnostic capabilities in lung cancer and can be indicative of survival outcomes [66]. Circular RNA obtained from serum exosomes (circ_0000735) has been shown to be involved in NSCLC progression and shows the possibility of its application as a diagnostic marker for NSCLC [67].

From the above references, it is evident that EV proteins and their cargo, including different types of RNA, can function as diagnostic biomarkers for lung cancer. This will certainly fuel research endeavors focusing on the development of novel biosensing platforms targeting different components of EVs [68]. Recently, a considerable number of studies have revealed the development of biosensing platforms for EVs and EV cargo in the context of lung cancer. A lateral flow aptamer assay targeting the CD63 membrane protein of exosomes has been developed for the detection of lung cancer [69]. A surface plasmon resonance technology-based sensor was developed to detect cancerous exosomes for differentiating lung cancer patients from healthy individuals. This sensor showed excellent clinical applicability [70]. Rapid advancements in sensing technologies that will address challenges like heterogeneity of EVs would pave the way for taking advantage of EV-based liquid biopsy [71].

## 5. Two Critical Factors for EV-Based Liquid Biopsies

Extensive research has suggested that EVs serve as valuable diagnostic biomarkers. When considering EVs as biomarkers, two critical factors must be considered: the effective isolation of EVs and the selection of an appropriate analysis method [72].

### 5.1. Isolation Techniques for EVs

**Figure 4:**
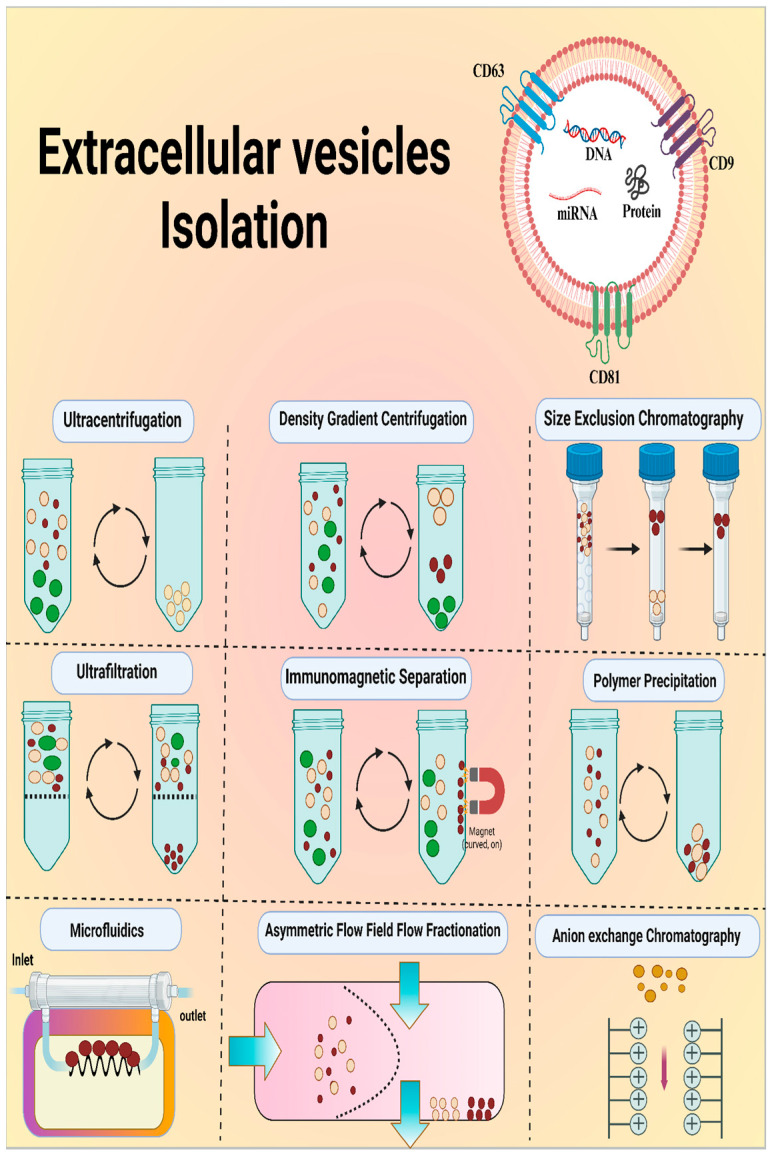
Labeled diagram of EV and illustration of common techniques for EV isolation.

The isolation of EVs is essential for fundamental scientific studies and practical medical applications. However, this is a challenging task. EVs are very similar in physical and chemical properties to other biological components, such as lipoproteins and protein clusters. Different methods for isolating EVs can significantly affect their number and purity, which can affect further analysis. Therefore, choosing an appropriate isolation technique is critical for ensuring the quality of research [73]. The most common techniques for EV isolation are shown in Figure 4, and the strengths and weaknesses of these techniques are tabulated in Table 3.

Ultracentrifugation (UC) is a common method used to isolate EVs. It works by spinning a sample at a high speed to separate particles based on their size. Different centrifugation steps remove larger objects, such as cells and debris, while isolating EVs through high-speed ultracentrifugation. The protocol most frequently followed was that of Théry et al. for the isolation of EVs through ultracentrifugation. Briefly, in step one, the sample was exposed to low-speed centrifugation at 300× *g* for 10 min to eliminate dead cells. In step two, centrifugation was performed at a medium speed of 2000× *g* for 15 min to eliminate cell debris. Step three involves high-speed centrifugation at 10,000× *g* for 30 min to eliminate large vesicles. Step four includes filtration through a 0.22 μm filter. Step five included super-high-speed ultracentrifugation at 100,000× *g* for 120 min. Step six included resuspension in PBS for subsequent ultracentrifugation. Finally, the EV pellet can be used immediately or stored at −80 °C for future use. However, UC suffers from discrepancies, such as the co-isolation of impurities with EVs, thereby compromising purity [74].

Density gradient centrifugation (DGC) separates EVs based on their density using a layered medium (sucrose or iodixanol) and ultracentrifugation. This method yields high-purity EVs that are ideal for functional assays; however, it can be slow and may recover fewer EVs owing to multiple centrifugation steps [75].

Size-Exclusion Chromatography (SEC) separates EVs by size using porous beads in a column. Smaller molecules flow faster, leaving EVs behind. This method is gentle for EVs and yields high-purity samples. However, it may dilute the EV samples and raise the requirement for post-isolation concentration steps [76].

Polymer precipitation, adapted from viral purification, uses PEG to precipitate EVs based on their physical properties. PEG is hydrophilic in nature and attracts water, causing EVs to aggregate owing to its reduced solubility. This method is efficient for large volumes and yields many EVs but also captures other proteins and particles, resulting in a lower purity of the EV sample [77].

Ultrafiltration (UF) uses pressure to quickly concentrate EVs from large volumes based on their molecular size. While efficient, it might miss smaller EVs and capture similar-sized proteins, making it ideal for high-throughput applications where moderate purity is sufficient [78].

Anion Exchange Chromatography (AEC) isolates EVs based on their negative charges. EVs bind to a column packed with charged resins, while other particles are washed through. The samples were pre-cleared using low-speed ultracentrifugation prior to the isolation of EVs using this method. EVs were obtained by altering the salt concentration and pH levels. This method yields high-purity EVs, but can be complex and time-consuming, requiring careful setup to avoid EV loss due to unwanted interactions with the column matrix [79].

Asymmetrical Flow Field-Flow Fractionation (AF4), a separation technique, uses a special channel lined with a semi-permeable membrane with a set size exclusion limit and a cross flow at 90 degrees to the sample flow direction to allow particles segregation thereby, enabling high-resolution isolation of EVs based on size. Unlike other methods, it avoids a stationary phase, which can damage EVs. This allows AF4 to gently isolate EVs, while providing information about their size and heterogeneity. However, its complexity and required equipment limit its wider use [80].

Affinity-based methods isolate EVs with high purity by targeting specific molecules on their surfaces, such as antibodies that target antigens. Typically, antibodies, aptamers, lipid moieties, peptides, or glucan-based elements are employed to separate EVs. This leads to very pure EV samples, but may recover fewer EVs and potentially alter their properties. Additionally, these methods can be expensive and difficult to scale up for large-scale applications [81].

Owing to their enormous impact on EV-based platforms, new techniques for EV isolation have seen major improvements. It is anticipated that in the future, we will be able to develop cutting-edge advanced techniques for EV isolation that can help us unlock the full potential of EVs as a source of liquid biopsy.

**Table 3:** Strengths and weaknesses of common isolation techniques for EVs [82,83,84,85,86,87].

Isolation Technique	Strengths	Weaknesses
Ultracentrifugation	Widely employed Purity is high	Need skilled operator Low scalability Costly & low yield
Density Gradient Centrifugation	Excellent purity Diverse samples types	Skilled operator Time consuming Not expandable Low yield
Size Exclusion Chromatography	Ubiquitous applications Limits contaminations Available commercially	Contaminant co-isolation Low yield
Ultrafiltration	High yield Scalable Diverse applicability	Damage the EVs Medium purity Clogging of membrane
Precipitation	Cost effective Quick High yield	Low purity High contamination risk
Asymmetrical Flow Field-Flow Fractionation	Quick and effective High-yield Readily expandable	Highs priced technology Intensive optimization
Tangential Flow Filtration	Large processing capacity Expandable Limited sample deterioration	Expensive apparatus Chance of pore clogging Co-isolation of contaminants
Anion Exchange Chromatography	Gentle handling Reliability No special tool requirements	Inefficient with complicated biofluids Laborious optimization
Immunoaffinity	Ultra cleans EVs Selective isolation of EV types No specialized tools needed	Narrow scope of scalability Damaging elution process Antibodies challenges
Microfluidic Platform	Dynamic capture and analysis Reduced sample volume Improved purity	Equipment expenses Expansion difficulties Consistency challenges

### 5.2. Analysis Techniques for EVs

**Figure 5:**
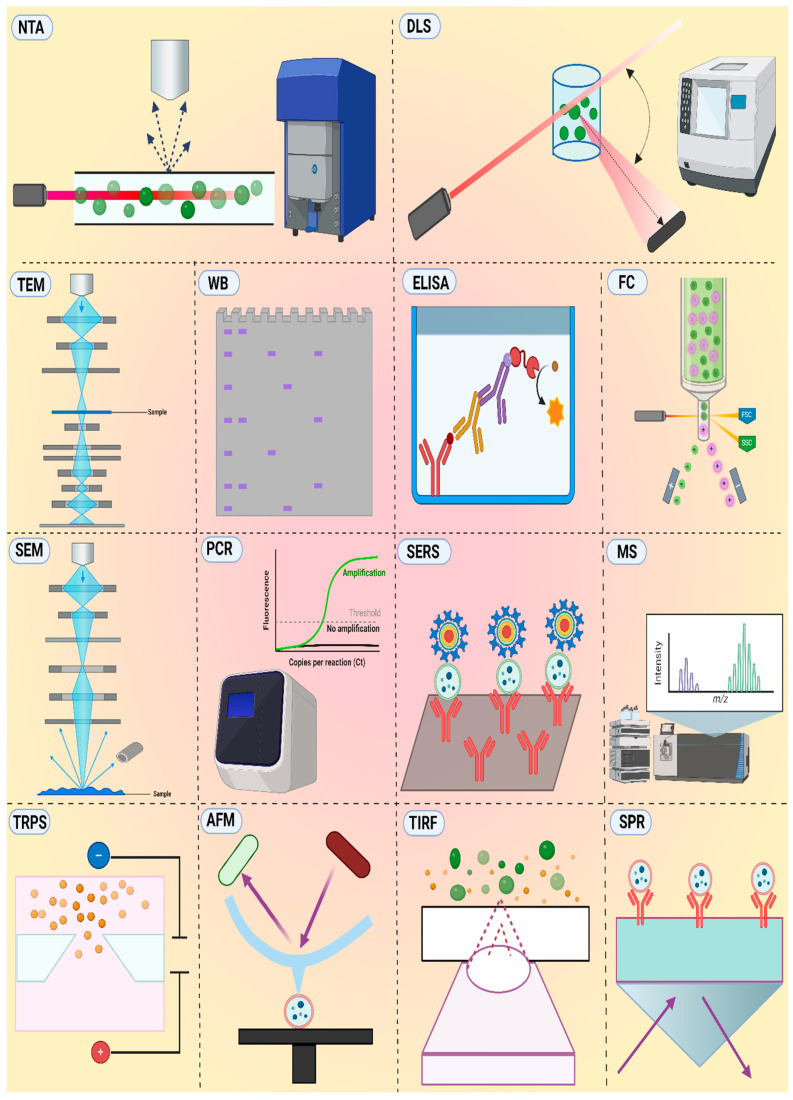
Various methods for EV analysis include (“NTA: Nanoparticle Tracking Analysis; AFM: Atomic Force Microscopy; TRPS: Tunable Resistive Pulse Sensing; SERS: Surface-Enhanced Raman Scattering; SEM: Scanning Electron Microscopy; TEM: Transmission Electron Microscopy; DLS: Dynamic Light Scattering; MS: Mass Spectrometry; PCR: Polymerase Chain Reaction; FC: Flow Cytometry; WB: Western Blotting; SPR: Surface Plasmon Resonance; ELISA: Enzyme-Linked Immunosorbent Assay; TIRF: Total Internal Reflection Fluorescence”).

Tools that provide an accurate analysis of EVs for the detection of diseases with clinical utility are highly valuable. The most common techniques for the analysis of EVs are shown in Figure 5, and their strengths and weaknesses are tabulated in Table 4. EVs’ size, complex composition, and heterogeneity pose challenges in their analysis with clinical prospects. Scientists are meticulously attempting to develop methodologies to study the biophysical and biochemical properties of EVs. Techniques that analyze EVs’ morphology, charge, size, and concentration provide insights into their physical nature. Techniques that study EV-specific proteins, nucleic acids, and other components provide insights into the biochemical composition of EVs [72]. Nanoparticle Tracking Analysis (NTA) is a commonly employed technique that uses light scattering to track and measure the size and concentration of EVs in real time. NTA involves a simple sample preparation step; however, the instrument is costly, cannot differentiate EVs from other nanoparticles, and cannot provide detailed morphological information [88]. NTA utility is encumbered by challenges regarding reproducibility and sensitivity when applied to EV analysis.

Atomic Force Microscopy (AFM) provides intricate high-resolution images of EVs under various environmental conditions, allowing the visualization of EV surface features and size determination. AFM employs a mechanical cantilever to scan the surfaces of EV adsorbed on mica or glass slides. The surface topology of EVs can cause deflection, providing information about the surface features and size of EVs. However, AFM requires specialized tips and expertise, and its slow analysis time limits its throughput [89]. Tunable Resistive Pulse Sensing (TRPS) detects the alteration in electrical current when an individual EV traverses a nanopore. It is valued for its high sensitivity and ability to differentiate EVs based on their size and charge. However, the need for specialized equipment and susceptibility to clogging by impurities hinder its widespread application [90]. Surface-Enhanced Raman Scattering (SERS) amplifies the Raman signal of molecules adhered to metal surfaces by exploiting the interaction between light and nanoscale metallic structures. This technique can be employed, for example, for the detection of EVs. This technique offers a powerful solution for the highly sensitive and non-destructive analysis of EVs and their components but requires sophisticated instrumentation and specialized expertise [91]. Transmission electron microscopy (TEM) employs an electron beam to produce high-resolution images of EVs based on scattered and transmitted electrons, revealing their morphology and internal structures. Scanning electron microscopy (SEM) uses a fine electron beam to scan the sample surface, causing secondary electron emission collected by a specialized detector, and the signal is converted to an image on the screen. While offering valuable information, both techniques require complex sample preparation and fixation steps, potentially altering the native state of EVs.

Dynamic Light Scattering (DLS) is also referred to as quasi-elastic light scattering, which utilizes the variation in scattered light to determine the size distribution of EVs by analyzing the Brownian movement of particles in suspension. This method offers a rapid, non-invasive alternative tool with minimal sample preparation for measuring EV size distribution; however, DLS cannot distinguish EVs from other similarly sized particles. DLS can provide information about EVs’ size and charge but cannot provide information about their concentration [92].

Mass Spectrometry (MS) serves as an effective analytical technique for examining complex samples and categorizing them according to their mass-to-charge ratio. This method has been employed to identify and characterize the protein, lipid, and nucleic acid content of EVs. Using this method, a vast array of information can be obtained; however, it requires complex instrumentation and specialized data analysis skills [93]. Polymerase chain reaction (PCR) is a state-of-the-art technique with a wide range of applications in nucleic acid detection. Various types of PCR have been successfully utilized for the analysis of nucleic acid cargo in EVs [94].

Another technique, Flow Cytometry (FC), has shown potential for the multiparametric analysis of the physical and biochemical attributes of EVs. It utilizes a light-scattering technique to acquire information about particle size and multicolor fluorescent markers to detect the expression of specific genes, proteins, or particle concentrations. Standard FC instruments, however, are not sensitive enough to identify EVs with dimensions less than 300–500 nm. As a result, advanced machines, such as NanoFCM, are marketed every year, which can perform multiparametric analysis of particles as small as 40 nm, but are costly [95]. Another frequently employed method for analyzing the protein composition of EVs is Western blotting (WB). The working principle involves the treatment of purified EVs with a solution that breaks down their structure (denaturants) while protecting proteins (protease inhibitors). Subsequently, the proteins underwent size-based separation using sodium dodecyl sulfate-polyacrylamide gel electrophoresis. Then, the separated proteins were transferred onto a blotting membrane to enable visualization. Incubated overnight with primary antibodies that work as recognition tags to target and bind to proteins of interest on the membrane. After washing, the sections were incubated with secondary antibodies for some hours. Finally, through an enhanced chemiluminescence reaction, the captured proteins of EVs were visualized. However, WB requires specific antibodies or primers, potentially unsuitable to provide a broader picture.

Surface Plasmon Resonance (SPR) is an optical biosensing tool that uses light to detect material properties by measuring small changes in the refractive index. Recently, scientists have used SPR to study EVs [96]. The workflow is simple, with molecules that attract EVs, such as antibodies or aptamers, adhered to the metal surface. After binding the EVs, changes in the refractive index were measured to provide a broader picture for the analysis of EVs. The analysis of fluorescent molecules or nanoparticles is conducted using the Total Internal Reflection Fluorescence (TIRF) method, which involves observing their fluorescence after excitation through total internal reflection. Florescence spots and their intensities were measured for estimated quantification [97]. Enzyme-Linked Immunosorbent Assay (ELISA) is a widely applicable technique that utilizes a solid phase carrying a primary agent, for instance, specific antibodies for capturing EVs or EV lysates and secondary agents for signals to quantify EVs. ELISA has already entered the EV research market with numerous specialized kits based on its principle [98]. However, ELISA also has to overcome issues such as laborious operation, the use of expensive antibodies, and the involvement of numerous washing and incubation steps.

**Table 4:** Strengths and weaknesses of common techniques for the analysis of EVs [85,99,100,101].

Technique	Strengths	Weaknesses
TEM	Fine precision Low sample consumption	Limited sample capacity Extensive sample processing
SEM	Easier sample handling than TEM Small sample requirement	Low image quality than TEM Sample damage during drying Coating can mask sample details
AFM	Offers biophysical information Analyze in physiological conditions	Limited sample capacity Need skilled personnel
NTA	Quantifies particle size and abundance. Enables live observation of particles Differentiates by particle size	Limited to particles ≥ 30 nm Extensive sample pretreatment
DLS	Few steps needed for analysis Expedited size determination	Insufficient for particle count Heterogeneous sizing challenge
TRPS	Measures particle size and abundance Customizable for diverse particles	Prone to particle clogging Influenced by conditions
WB	Identify specific markers on EVs Supports multi-protein profiling	Labor-intensive Poor sensitivity
ELISA	Quantifies protein content Enables high-throughput analysis	Risk of antibody cross-reactivity Inconsistent sensitivity
MS	Comprehensive analysis of diverse biomolecules Detects molecules at minimal levels	Requires bioinformatic expertise Labor-intensive processing Costly equipment and upkeep
PCR	Detects trace levels of nucleic acids Precisely measure nucleic acid	Sensitive to nucleic acid purity Susceptible to contamination
TIRF	Enhanced data accuracy Visualizes EV–cell interactions	Narrow observational range Alignment & calibration critical
FC	Offers multiparametric analysis of EVs High-speed analysis Reveals EV diversity	Can not analyze tiny EVs Non-specific interactions Costly instrumentation
SPR	Quantifies minute concentrations No labeling or staining required	Refractive index sensitivity Requires Sensor optimization
SERS	Detect trace levels of analytes Multi-component analysis	Poor repeatability of results Background interference

Current detection methods for EVs still have to overcome issues such as low sensitivity, low specificity, and cumbersome operation. Thus, there is a need to create advanced detection platforms that are user-friendly, highly sensitive, and specific. In conclusion, a multitude of EV analysis methods exist, each with its own strengths and limitations [101]. The optimal approach depends on the research question and desired information. Considering the capabilities and limitations of these techniques, researchers can effectively unlock the diagnostic capabilities of EVs and gain deeper insights into their optimal utilization in liquid biopsies of various malignancies, including lung cancer.

## 6. Challenges

For early detection, management, and monitoring of lung cancer outcomes, liquid biopsy serves as an exceptionally sophisticated diagnostic method [102]. EV-based diagnostic interventions have been widely reported by researchers, but many obstacles still need to be overcome to translate scientific discoveries into clinical practice [103]. A significant challenge in the field of EV research is the lack of standard methods for EV isolation. There is a huge variability in the methods used to isolate exosomes, including ultracentrifugation, precipitation, immunoaffinity capture, and microfluidic devices. Each method can preferentially isolate different EV subpopulations, leading to variations in their composition and function. The choice of isolation method can significantly affect the detection of specific exosomal biomarkers, potentially affecting the accuracy of diagnostic tests. Isolation techniques sometimes fail to address the heterogeneity of the EV population, which may interfere with biomarker analysis. The purity of isolated exosomes is sometimes compromised by cellular debris, which affects the reliability of downstream analyses. As there is no standardized protocol to date, a comparison of the results of different studies is not possible. It is also pertinent to establish quality control measures, such as electron microscopy and flow cytometry, to ensure the purity and integrity of isolated EVs [104].

Other challenging factors that need to be carefully considered prior to taking full advantage of EV-based clinically proven diagnostic value are the selection of detection techniques, biomarker expression levels, sample variability, and potential sources of interference [105]. The absence of large-scale validation in clinical studies is a critical point that requires attention to harness the potential of EV-based methods for lung cancer detection. At present, the sample sizes in relevant studies are insufficient, data are limited, and validation periods are often too short to draw definitive conclusions. Moreover, the majority of studies have primarily focused on the specificity and sensitivity of exosome detection systems without adequately addressing other crucial aspects, such as consistency, reproducibility, accuracy, reference ranges, and minimum detection limits, which are essential for comprehensive testing [106]. Therefore, ongoing research in this area is essential to fully realize the potential of EV-based diagnostics for lung cancer detection and prognosis.

## 7. Future Prospects

EVs as potential candidates for liquid biopsy have shown outstanding prospects, given that cancer cells actively release these vesicles into bodily fluids, at approximately 20,000 EVs within 48 h [107]. EVs carry various biologically important molecules from secreted cells. These molecules, such as miRNA, mRNA, and DNA, are well protected by the double phospholipid layer membrane structure of EVs. The advantage of exosomes lies in their abundance. ctDNA is mostly produced during cancer cell death; however, EVs are continuously secreted by living cancer cells and can provide exact insight into the progression of tumor development in real time. However, a huge chunk of research exploring the influence, mechanism of action, and biology of EVs is still in its infancy and has been conducted in vitro or in vivo. Therefore, research exploring EV-based interventions in humans with large-scale clinical validation is highly recommended. Research involving EV-based biomarker development must address some issues prior to making it commercially applicable in clinical settings. First, biomarker research needs to be validated by passing it through multiple stages, including preclinical studies, pilot clinical trials, and large-scale clinical trials. Future studies should employ robust statistical testing to demonstrate the statistical significance and clinical relevance of these biomarkers. The design of these studies should ensure generalizability by testing them in diverse populations. An important aspect for future studies focusing on EV-based biomarker development is to carefully consider pre-analytical factors, such as the method of choice for EV isolation and analysis [108], storage conditions, and processing conditions that can influence the accuracy of the biomarker [109]. Biomarkers can be indicative of patient outcomes or can aid in informing treatment decisions, as biomarkers can be implemented for large-scale screening; therefore, cost-effectiveness should also be one of the objectives of biomarker development research strategies. For a biomarker to pass through a lengthy regulatory process, it must demonstrate its safety, efficacy, and quality [110]. Multidisciplinary research efforts involving researchers, clinicians, regularity agencies, and industry partners are highly recommended for the development of clinically useful EV biomarkers [111].

## 8. Conclusions

Lung cancer is a major challenge for global healthcare, causing the death of millions of people every year across the globe. Rapid progress has been made in exploring lung tumor biology and therapeutic interventions, but research aimed at the development of efficient and sensitive diagnostic technologies still needs to be improved. Although there has been an overreliance on conventional tumor biopsy for lung cancer diagnosis and subtyping for a long time, it cannot overlook the associated limitations. Tissue biopsy provides very limited information about tumor burden and tumor heterogeneity despite being invasive, risky, costly, and inaccessible in most cases. The limitations of current diagnostic approaches necessitate novel interventions for the diagnosis of lung cancer. Increasing interest has been seen in the development of non-invasive techniques, such as liquid biopsy, that rely on body fluid for the analysis of cancer-specific biomarkers, thereby offering less invasive diagnosis and prognosis tools. Liquid biopsy using EVs is a promising new method with fundamental clinical advantages. EVs can be obtained non-invasively, provide real-time assessment of the tumor’s molecular status, and can be used to monitor disease progression and treatment. Given these potential applications, EVs can be distinctive biomarkers that can be utilized independently or in conjunction with other liquid biopsy methods. With the development of advanced techniques for EV isolation and analysis, cancer cell-derived EVs can transform cancer diagnostics, including lung cancer. The ubiquitous presence of EVs across body fluids is ideal for liquid biopsy, which relies on capturing EVs non-invasively from body fluids for analysis. Liquid biopsy with EVs is a revolutionizing approach because EVs have high clinical relevance in cancer diagnosis, monitoring of treatment response, and outcome forecasting. Consequently, EVs are at the forefront of advancements in diagnostic technologies for lung oncology.

## Figures and Tables

**Table 1:** Additional subtypes of EVs.

Subtype	Size	Origination	Markers	Ref.
Exosomes	30–150 nm	Late endosomal through MVBs	CD9, CD63, Tsg101, CD81, ALIX, HSP70	[31]
Microvesicles	100–1000 nm	Directly through plasma membrane budding	Integrins, Selectins, CD40, tissue factor	[32]
Apoptotic bodies	100–5000 nm	Through programmed cell death	Annexin V, C3b, thrombospondin, Annexin A1, histone coagulation factor	[33]
Exomeres	≤50 nm	Through cleavage of cytoplasmic extension	TGFBI, ENO1 and GPC1	[34]
Migrasomes	500–3000	Through bifurcation of retraction fibers during cell migration	Tspan4, CD63, Annexin A1	[35]
Oncosomes	1000–10,000	Through cancer cell amoeboid movement	Cav-1 or ADP ribosylation factor 6	[36]
Supermeres	35–50	Not explored	TGFBI, ACE2, PCSK9, miR-1246, MET, GPC1 and AGO2.	[37]

**Table 2:** Extracellular vesicles and their cargo for the diagnosis of lung cancer.

Source	EV-Based Biomarker	Utility	Ref.
Plasma	NY-ESO-1	Prognostic	[44]
Serum	PD-L1	Diagnosis	[45]
Urine	LRG1	Diagnosis	[46]
Serum	PD-L1	Prognosis	[47]
Plasma	EpCam	Diagnosis and prognosis	[48]
Serum	EGFR	Diagnosis	[49]
plasma	CD151, CD171, and tetraspanin 8	Diagnosis	[44]
Plasma	CD63, CD9, CD81	Diagnosis and prognosis	[50]
Plasma	miR-19-3p, miR-221-3p, and miR-21-5p	Diagnosis	[51]
Plasma	miR-21 and miR-4257	Prognosis	[52]
Pleural effusion	miR-200	Diagnosis	[53]
Saliva	miR-92b-5p	Diagnosis	[54]
Urine and saliva	miRNA-205	Diagnosis	[55]
Serum	miR-125b-5p	Diagnosis	[56]
Plasma	miR-30b/30c	Diagnosis	[57]

## References

[1] Qu H., Zhu M., Shan C., Ji X., Ji G., Zhang W., Zhang H., Chen B. (2023). Prevalence, diagnosis, and treatment of chronic obstructive pulmonary disease in a hospitalized lung cancer population: A single center study. J. Thorac. Dis..

[2] Leiter A., Veluswamy R.R., Wisnivesky J.P. (2023). The global burden of lung cancer: Current status and future trends. Nat. Rev. Clin. Oncol..

[3] Elshoeibi A.M., Elsayed B., Kaleem M.Z., Elhadary M.R., Abu-Haweeleh M.N., Haithm Y., Krzyslak H., Vranic S., Pedersen S. (2023). Proteomic Profiling of Small-Cell Lung Cancer: A Systematic Review. Cancers.

[4] Mehta A., Barreto G. (2018). Non-invasive approaches for lung cancer diagnosis. Indian J. Thorac. Cardiovasc. Surg..

[5] Meriggi F. (2024). Second-Line Treatment Options for Small-Cell Lung Cancer: A Light at The End of the Tunnel. Cancers.

[6] Bray F., Laversanne M., Sung H., Ferlay J., Siegel R.L., Soerjomataram I., Jemal A. (2024). Global cancer statistics 2022: GLOBOCAN estimates of incidence and mortality worldwide for 36 cancers in 185 countries. CA Cancer J. Clin..

[7] Zhao D., Lu J., Zeng W., Zhang C., You Y. (2024). Changing trends in disease burden of lung cancer in China from 1990–2019 and following 15-year prediction. Curr. Probl. Cancer.

[8] Sharma R. (2022). Mapping of global, regional and national incidence, mortality and mortality-to-incidence ratio of lung cancer in 2020 and 2050. Int. J. Clin. Oncol..

[9] Ramadan M., Alhusseini N., Samhan L., Samhan S., Abbad T. (2023). Tobacco control policies implementation and future lung cancer incidence in Saudi Arabia. A population-based study. Prev. Med. Rep..

[10] Zhang J., Wang S., Zhou Z., Lei C., Yu H., Zeng C., Xia X., Qiao G., Shi Q. (2023). Unpleasant symptoms of immunotherapy for people with lung cancer: A mixed-method study. Int. J. Nurs. Stud..

[11] Kops S.E., Heus P., Korevaar D.A., Damen J.A., Idema D.L., Verhoeven R.L., Annema J.T., Hooft L., van der Heijden E.H. (2023). Diagnostic yield and safety of navigation bronchoscopy: A systematic review and meta-analysis. Lung Cancer.

[12] Visser E., Genet S.A.A.M., de Kock R.P.P.A., van den Borne B.E.E.M., Youssef-El Soud M., Belderbos H.N.A., Stege G., de Saegher M.E.A., van’t Westeinde S.C., Brunsveld L. (2023). Liquid biopsy-based decision support algorithms for diagnosis and subtyping of lung cancer. Lung Cancer.

[13] Chinnappan R., Mir T.A., Alsalameh S., Makhzoum T., Alzhrani A., Alnajjar K., Adeeb S., Al Eman N., Ahmed Z., Shakir I. (2023). Emerging Biosensing Methods to Monitor Lung Cancer Biomarkers in Biological Samples: A Comprehensive Review. Cancers.

[14] Jalal A.H., Sikder A.K., Alam F., Samin S., Rahman S.S., Khan M.M.A., Siddiquee M.R. (2021). Early diagnosis with alternative approaches: Innovation in lung cancer care. Shanghai Chest.

[15] Silvestri G.A., Goldman L., Tanner N.T., Burleson J., Gould M., Kazerooni E.A., Mazzone P.J., Rivera M.P., Doria-Rose V.P., Rosenthal L.S. (2023). Outcomes from more than 1 million people screened for lung cancer with low-dose CT imaging. Chest.

[16] Ledda R.E., Funk G.-C., Sverzellati N. (2024). The pros and cons of lung cancer screening. Eur. Radiol..

[17] Zarinshenas R., Amini A., Mambetsariev I., Abuali T., Fricke J., Ladbury C., Salgia R. (2023). Assessment of barriers and challenges to screening, diagnosis, and biomarker testing in early-stage lung cancer. Cancers.

[18] De Margerie-Mellon C., Chassagnon G. (2023). Artificial intelligence: A critical review of applications for lung nodule and lung cancer. Diagn. Interv. Imaging.

[19] Medhin L.B., Beasley A.B., Warburton L., Amanuel B., Gray E.S. (2023). Extracellular vesicles as a liquid biopsy for melanoma: Are we there yet?. Semin. Cancer Biol..

[20] Li L., Jiang H., Zeng B., Wang X., Bao Y., Chen C., Ma L., Yuan J. (2024). Liquid biopsy in lung cancer. Clin. Chim. Acta.

[21] Bamankar S., Londhe V.Y. (2023). The rise of extracellular vesicles as new age biomarkers in cancer diagnosis: Promises and pitfalls. Technol. Cancer Res. Treat..

[22] Zhou Q., Niu X., Zhang Z., O’Byrne K., Kulasinghe A., Fielding D., Möller A., Wuethrich A., Lobb R.J., Trau M. (2024). Glycan Profiling in Small Extracellular Vesicles with a SERS Microfluidic Biosensor Identifies Early Malignant Development in Lung Cancer. Adv. Sci..

[23] Ilié M., Hofman P. (2016). Pros: Can tissue biopsy be replaced by liquid biopsy?. Transl. Lung Cancer Res..

[24] Thenuwara G., Curtin J., Tian F. (2023). Advances in diagnostic tools and therapeutic approaches for gliomas: A comprehensive review. Sensors.

[25] Antoniou S., Gaude E., Ruparel M., Van Der Schee M., Janes S., Rintoul R., Group L.R. (2019). The potential of breath analysis to improve outcome for patients with lung cancer. J. Breath Res..

[26] Mahuron K.M., Fong Y. (2024). Applications of liquid biopsy for surgical patients with cancer: A review. JAMA Surg..

[27] Shegekar T., Vodithala S., Juganavar A. (2023). The emerging role of liquid biopsies in revolutionising cancer diagnosis and therapy. Cureus.

[28] Cui S., Cheng Z., Qin W., Jiang L. (2018). Exosomes as a liquid biopsy for lung cancer. Lung Cancer.

[29] Davidson S.M., Boulanger C.M., Aikawa E., Badimon L., Barile L., Binder C.J., Brisson A., Buzas E., Emanueli C., Jansen F. (2023). Methods for the identification and characterization of extracellular vesicles in cardiovascular studies: From exosomes to microvesicles. Cardiovasc. Res..

[30] Welsh J.A., Goberdhan D.C.I., O’Driscoll L., Buzas E.I., Blenkiron C., Bussolati B., Cai H., Di Vizio D., Driedonks T.A.P., Erdbrügger U. (2024). Minimal information for studies of extracellular vesicles (MISEV2023): From basic to advanced approaches. J. Extracell. Vesicles.

[31] Kalluri R., LeBleu V.S. (2020). The biology, function, and biomedical applications of exosomes. Science.

[32] Boysen J., Nelson M., Magzoub G., Maiti G.P., Sinha S., Goswami M., Vesely S.K., Shanafelt T.D., Kay N.E., Ghosh A.K. (2017). Dynamics of microvesicle generation in B-cell chronic lymphocytic leukemia: Implication in disease progression. Leukemia.

[33] Li G., Chen T., Dahlman J., Eniola-Adefeso L., Ghiran I.C., Kurre P., Lam W.A., Lang J.K., Marbán E., Martín P. (2023). Current challenges and future directions for engineering extracellular vesicles for heart, lung, blood and sleep diseases. J. Extracell. Vesicles.

[34] Greening D.W., Simpson R.J. (2018). Understanding extracellular vesicle diversity–current status. Expert Rev. Proteom..

[35] Zhang X., Yao L., Meng Y., Li B., Yang Y., Gao F. (2023). Migrasome: A new functional extracellular vesicle. Cell Death Discov..

[36] Shishido S.N., Lin E., Nissen N., Courcoubetis G., Suresh D., Mason J., Osipov A., Hendifar A.E., Lewis M., Gaddam S. (2024). Cancer-related cells and oncosomes in the liquid biopsy of pancreatic cancer patients undergoing surgery. NPJ Precis. Oncol..

[37] Zhang Q., Jeppesen D.K., Higginbotham J.N., Franklin J.L., Coffey R.J. (2023). Comprehensive isolation of extracellular vesicles and nanoparticles. Nat. Protoc..

[38] Liu C., Xiang X., Han S., Lim H.Y., Li L., Zhang X., Ma Z., Yang L., Guo S., Soo R. (2022). Blood-based liquid biopsy: Insights into early detection and clinical management of lung cancer. Cancer Lett..

[39] Ruzycka-Ayoush M., Prochorec-Sobieszek M., Cieszanowski A., Glogowski M., Szumera-Cieckiewicz A., Podgorska J., Targonska A., Sobczak K., Mosieniak G., Grudzinski I.P. (2024). Extracellular Vesicles as Next-Generation Biomarkers in Lung Cancer Patients: A Case Report on Adenocarcinoma and Squamous Cell Carcinoma. Life.

[40] Rahimian S., Najafi H., Afzali B., Doroudian M. (2024). Extracellular Vesicles and Exosomes: Novel Insights and Perspectives on Lung Cancer from Early Detection to Targeted Treatment. Biomedicines.

[41] Wang Y., Shen C., Zeng X., Xiong Y., Li K., Huang K., Chen P. (2024). Tandem hybridization chain reaction and selective coordination enable fluorescence detection of exosomes in lung cancer. Sens. Actuators B Chem..

[42] Lim S., Ha Y., Lee B., Shin J., Rhim T. (2024). Calnexin as a dual-role biomarker: Antibody-based diagnosis and therapeutic targeting in lung cancer. BMB Rep..

[43] Xu F., Luo S., Lu P., Cai C., Li W., Li C. (2024). Composition, functions, and applications of exosomal membrane proteins. Front. Immunol..

[44] Sandfeld-Paulsen B., Aggerholm-Pedersen N., Bæk R., Jakobsen K.R., Meldgaard P., Folkersen B.H., Rasmussen T.R., Varming K., Jørgensen M.M., Sorensen B.S. (2016). Exosomal proteins as prognostic biomarkers in non-small cell lung cancer. Mol. Oncol..

[45] Khan A., Di K., Khan H., He N., Li Z. (2022). Rapid Capturing and Chemiluminescent Sensing of Programmed Death Ligand-1 Expressing Extracellular Vesicles. Biosensors.

[46] Li Y., Zhang Y., Qiu F., Qiu Z. (2011). Proteomic identification of exosomal LRG1: A potential urinary biomarker for detecting NSCLC. Electrophoresis.

[47] Kim D.H., Kim H., Choi Y.J., Kim S.Y., Lee J.-E., Sung K.J., Sung Y.H., Pack C.-G., Jung M.-k., Han B. (2019). Exosomal PD-L1 promotes tumor growth through immune escape in non-small cell lung cancer. Exp. Mol. Med..

[48] Yoh K.E., Lowe C.J., Mahajan S., Suttmann R., Nguy T., Reichelt M., Yang J., Melendez R., Li Y., Molinero L. (2021). Enrichment of circulating tumor-derived extracellular vesicles from human plasma. J. Immunol. Methods.

[49] Yamashita T., Kamada H., Kanasaki S., Maeda Y., Nagano K., Abe Y., Inoue M., Yoshioka Y., Tsutsumi Y., Katayama S. (2013). Epidermal growth factor receptor localized to exosome membranes as a possible biomarker for lung cancer diagnosis. Pharmazie.

[50] Sandfeld-Paulsen B., Jakobsen K.R., Bæk R., Folkersen B.H., Rasmussen T.R., Meldgaard P., Varming K., Jørgensen M.M., Sorensen B.S. (2016). Exosomal proteins as diagnostic biomarkers in lung cancer. J. Thorac. Oncol..

[51] Zhou X., Wen W., Shan X., Zhu W., Xu J., Guo R., Cheng W., Wang F., Qi L.-W., Chen Y. (2017). A six-microRNA panel in plasma was identified as a potential biomarker for lung adenocarcinoma diagnosis. Oncotarget.

[52] Dejima H., Iinuma H., Kanaoka R., Matsutani N., Kawamura M. (2017). Exosomal microRNA in plasma as a non-invasive biomarker for the recurrence of non-small cell lung cancer. Oncol. Lett..

[53] Hydbring P., De Petris L., Zhang Y., Brandén E., Koyi H., Novak M., Kanter L., Hååg P., Hurley J., Tadigotla V. (2018). Exosomal RNA-profiling of pleural effusions identifies adenocarcinoma patients through elevated miR-200 and LCN2 expression. Lung Cancer.

[54] Liu M., Yu X., Bu J., Xiao Q., Ma S., Chen N., Qu C. (2023). Comparative analyses of salivary exosomal miRNAs for patients with or without lung cancer. Front. Genet..

[55] Zhou P., Lu F., Wang J., Wang K., Liu B., Li N., Tang B. (2020). A portable point-of-care testing system to diagnose lung cancer through the detection of exosomal miRNA in urine and saliva. Chem. Commun..

[56] Zhang Z., Tang Y., Song X., Xie L., Zhao S., Song X. (2020). Tumor-derived exosomal miRNAs as diagnostic biomarkers in non-small cell lung cancer. Front. Oncol..

[57] Rolfo C., Chacartegui J., Giallombardo M., Alessandro R., Peeters M. (2016). 7P Exosomes isolated in plasma of non-small cell lung cancer patients contain microRNA related to the EGFR pathway: Proof of concept. J. Thorac. Oncol..

[58] Sha L., Bo B., Li J., Liu Q., Cao Y., Zhao J. (2024). Precise assessment of lung cancer-derived exosomes based on dual-labelled membrane interface. Chin. Chem. Lett..

[59] Luo S., Wu Y., Pan W., Zhong G., Situ B., Li B., Ye X., Jiang X., Li W., Zhang Y. (2023). An integrated magneto-fluorescent nanosensor for rapid and sensitive detection of tumor-derived exosomes. Sens. Actuators B Chem..

[60] Feng J., Jia L., Pan W., Fan Y., Guo J., Luo T., Liu C., Wang W., Zheng L., Li B. (2023). Rapid and efficient fluorescent aptasensor for PD-L1 positive extracellular vesicles isolation and analysis: EV-ANCHOR. Chem. Eng. J..

[61] Ren F., Fei Q., Qiu K., Zhang Y., Zhang H., Sun L. (2024). Liquid biopsy techniques and lung cancer: Diagnosis, monitoring and evaluation. J Exp Clin Cancer Res..

[62] Rao D.-Y., Huang D.-F., Si M.-Y., Lu H., Tang Z.-X., Zhang Z.-X. (2023). Role of exosomes in non-small cell lung cancer and EGFR-mutated lung cancer. Front. Immunol..

[63] Kan C.F.K., Unis G.D., Li L.Z., Gunn S., Li L., Soyer H.P., Stark M.S. (2021). Circulating Biomarkers for Early Stage Non-Small Cell Lung Carcinoma Detection: Supplementation to Low-Dose Computed Tomography. Front. Oncol..

[64] Zhang Q., Qin S., Peng C., Liu Y., Huang Y., Ju S. (2023). Circulating circular RNA hsa_circ_0023179 acts as a diagnostic biomarker for non-small-cell lung cancer detection. J. Cancer Res. Clin. Oncol..

[65] Cazzoli R., Buttitta F., Di Nicola M., Malatesta S., Marchetti A., Rom W.N., Pass H.I. (2013). microRNAs derived from circulating exosomes as noninvasive biomarkers for screening and diagnosing lung cancer. J. Thorac. Oncol..

[66] Yang B., Xin X., Cao X., Nasifu L., Nie Z., He B. (2024). The diagnostic and prognostic value of exosomal microRNAs in lung cancer: A systematic review. Clin. Transl. Oncol..

[67] Liu S., Wu X., Wang Y., Chen Y. (2024). Exosomal circ_0000735 contributes to non-small lung cancer malignant progression. J. Biochem. Mol. Toxicol..

[68] Cavallaro S., Hååg P., Sahu S.S., Berisha L., Kaminskyy V.O., Ekman S., Lewensohn R., Linnros J., Viktorsson K., Dev A. (2021). Multiplexed electrokinetic sensor for detection and therapy monitoring of extracellular vesicles from liquid biopsies of non-small-cell lung cancer patients. Biosens. Bioelectron..

[69] Yu Q., Zhao Q., Wang S., Zhao S., Zhang S., Yin Y., Dong Y. (2020). Development of a lateral flow aptamer assay strip for facile identification of theranostic exosomes isolated from human lung carcinoma cells. Anal. Biochem..

[70] Fan Y., Duan X., Zhao M., Wei X., Wu J., Chen W., Liu P., Cheng W., Cheng Q., Ding S. (2020). High-sensitive and multiplex biosensing assay of NSCLC-derived exosomes via different recognition sites based on SPRi array. Biosens. Bioelectron..

[71] Conteduca D., Brunetti G., Barth I., Quinn S.D., Ciminelli C., Krauss T.F. (2023). Multiplexed Near-Field Optical Trapping Exploiting Anapole States. ACS Nano.

[72] Wang Z., Zhou X., Kong Q., He H., Sun J., Qiu W., Zhang L., Yang M. (2024). Extracellular Vesicle Preparation and Analysis: A State-of-the-Art Review. Adv. Sci..

[73] Zhao Z., Wijerathne H., Godwin A.K., Soper S.A. (2021). Isolation and analysis methods of extracellular vesicles (EVs). Extracell. Vesicles Circ. Nucleic Acids.

[74] Théry C., Zitvogel L., Amigorena S. (2002). Exosomes: Composition, biogenesis and function. Nat. Rev. Immunol..

[75] Clos-Sansalvador M., Monguió-Tortajada M., Roura S., Franquesa M., Borras F.E. (2022). Commonly used methods for extracellular vesicles’ enrichment: Implications in downstream analyses and use. Eur. J. Cell Biol..

[76] Yang D., Zhang W., Zhang H., Zhang F., Chen L., Ma L., Larcher L.M., Chen S., Liu N., Zhao Q. (2020). Progress, opportunity, and perspective on exosome isolation-efforts for efficient exosome-based theranostics. Theranostics.

[77] Macías M., Rebmann V., Mateos B., Varo N., Perez-Gracia J.L., Alegre E., González Á. (2019). Comparison of six commercial serum exosome isolation methods suitable for clinical laboratories. Effect in cytokine analysis. Clin. Chem. Lab. Med. (CCLM).

[78] He L., Zhu D., Wang J., Wu X. (2019). A highly efficient method for isolating urinary exosomes. Int. J. Mol. Med..

[79] Pirolli N.H., Reus L.S.C., Mamczarz Z., Khan S., Bentley W.E., Jay S.M. (2023). High performance anion exchange chromatography purification of probiotic bacterial extracellular vesicles enhances purity and anti-inflammatory efficacy. Biotechnol. Bioeng..

[80] Bian J., Gobalasingham N., Purchel A., Lin J. (2023). The power of field-flow fractionation in characterization of nanoparticles in drug delivery. Molecules.

[81] Ströhle G., Gan J., Li H. (2022). Affinity-based isolation of extracellular vesicles and the effects on downstream molecular analysis. Anal. Bioanal. Chem..

[82] De Sousa K.P., Rossi I., Abdullahi M., Ramirez M.I., Stratton D., Inal J.M. (2023). Isolation and characterization of extracellular vesicles and future directions in diagnosis and therapy. WIREs Nanomed. Nanobiotechnol..

[83] Visan K.S., Wu L.-Y., Voss S., Wuethrich A., Möller A. (2023). Status quo of Extracellular Vesicle isolation and detection methods for clinical utility. Semin. Cancer Biol..

[84] Williams S., Fernandez-Rhodes M., Law A., Peacock B., Lewis M.P., Davies O.G. (2023). Comparison of extracellular vesicle isolation processes for therapeutic applications. J. Tissue Eng..

[85] Pallares-Rusiñol A., Bernuz M., Moura S.L., Fernández-Senac C., Rossi R., Martí M., Pividori M.I., Makowski G.S. (2023). Chapter Two-Advances in exosome analysis. Advances in Clinical Chemistry.

[86] Havers M., Broman A., Lenshof A., Laurell T. (2023). Advancement and obstacles in microfluidics-based isolation of extracellular vesicles. Anal. Bioanal. Chem..

[87] Omrani M., Beyrampour-Basmenj H., Jahanban-Esfahlan R., Talebi M., Raeisi M., Serej Z.A., Akbar-Gharalari N., Khodakarimi S., Wu J., Ebrahimi-Kalan A. (2024). Global trend in exosome isolation and application: An update concept in management of diseases. Mol. Cell. Biochem..

[88] Bachurski D., Schuldner M., Nguyen P.-H., Malz A., Reiners K.S., Grenzi P.C., Babatz F., Schauss A.C., Hansen H.P., Hallek M. (2019). Extracellular vesicle measurements with nanoparticle tracking analysis–An accuracy and repeatability comparison between NanoSight NS300 and ZetaView. J. Extracell. Vesicles.

[89] Parisse P., Rago I., Ulloa Severino L., Perissinotto F., Ambrosetti E., Paoletti P., Ricci M., Beltrami A.P., Cesselli D., Casalis L. (2017). Atomic force microscopy analysis of extracellular vesicles. Eur. Biophys. J..

[90] Maas S.L., Broekman M.L., de Vrij J. (2017). Tunable resistive pulse sensing for the characterization of extracellular vesicles. Exosomes Microvesicles Methods Protoc..

[91] Pang Y., Shi J., Yang X., Wang C., Sun Z., Xiao R. (2020). Personalized detection of circling exosomal PD-L1 based on Fe3O4@ TiO2 isolation and SERS immunoassay. Biosens. Bioelectron..

[92] Palmieri V., Lucchetti D., Gatto I., Maiorana A., Marcantoni M., Maulucci G., Papi M., Pola R., De Spirito M., Sgambato A. (2014). Dynamic light scattering for the characterization and counting of extracellular vesicles: A powerful noninvasive tool. J. Nanoparticle Res..

[93] Jimenez L., Yu H., McKenzie A.J., Franklin J.L., Patton J.G., Liu Q., Weaver A.M. (2019). Quantitative proteomic analysis of small and large extracellular vesicles (EVs) reveals enrichment of adhesion proteins in small EVs. J. Proteome Res..

[94] Jin Y., Chen K., Wang Z., Wang Y., Liu J., Lin L., Shao Y., Gao L., Yin H., Cui C. (2016). DNA in serum extracellular vesicles is stable under different storage conditions. BMC Cancer.

[95] Welsh J.A., Van Der Pol E., Arkesteijn G.J., Bremer M., Brisson A., Coumans F., Dignat-George F., Duggan E., Ghiran I., Giebel B. (2020). MIFlowCyt-EV: A framework for standardized reporting of extracellular vesicle flow cytometry experiments. J. Extracell. Vesicles.

[96] Wang X., Phan M.M., Sun Y., Koerber J.T., Ho H., Chen Y., Yang J. (2022). Development of an SPR-based binding assay for characterization of anti-CD20 antibodies to CD20 expressed on extracellular vesicles. Anal. Biochem..

[97] Martín-Cófreces N.B., Torralba D., Lozano-Prieto M., Fernández-Gallego N., Sánchez-Madrid F. (2021). TIRF microscopy as a tool to determine exosome composition. Stem Cell Renew. Cell-Cell Commun. Methods Protoc..

[98] Lee J., Kim H., Heo Y., Yoo Y.K., Han S.I., Kim C., Hur D., Kim H., Kang J.Y., Lee J.H. (2020). Enhanced paper-based ELISA for simultaneous EVs/exosome isolation and detection using streptavidin agarose-based immobilization. Analyst.

[99] Altıntaş O., Saylan Y. (2023). Exploring the versatility of exosomes: A review on isolation, characterization, detection methods, and diverse applications. Anal. Chem..

[100] Sonbhadra S., Mehak, Pandey L.M. (2023). Biogenesis, isolation, and detection of exosomes and their potential in therapeutics and Diagnostics. Biosensors.

[101] Zhang Q., Wang H., Liu Q., Zeng N., Fu G., Qiu Y., Yang Y., Yuan H., Wang W., Li B. (2024). Exosomes as Powerful Biomarkers in Cancer: Recent Advances in Isolation and Detection Techniques. Int. J. Nanomed..

[102] Palmieri M., Frullanti E. (2023). Different Liquid Biopsies for the Management of Non-Small Cell Lung Cancer in the Mutational Oncology Era. Med. Sci..

[103] Di Capua D., Bracken-Clarke D., Ronan K., Baird A.-M., Finn S. (2021). The Liquid Biopsy for Lung Cancer: State of the Art, Limitations and Future Developments. Cancers.

[104] Ospina A.V. (2024). Overview of the Role of Liquid Biopsy in Non-small Cell Lung Cancer (NSCLC). Clin. Oncol..

[105] Mousavi S.M., Amin Mahdian S.M., Ebrahimi M.S., Taghizadieh M., Vosough M., Sadri Nahand J., Hosseindoost S., Vousooghi N., Javar H.A., Larijani B. (2022). Microfluidics for detection of exosomes and microRNAs in cancer: State of the art. Mol. Ther.-Nucleic Acids.

[106] Lee K.W.A., Chan L.K.W., Hung L.C., Phoebe L.K.W., Park Y., Yi K.-H. (2024). Clinical Applications of Exosomes: A Critical Review. Int. J. Mol. Sci..

[107] Yu W., Hurley J., Roberts D., Chakrabortty S.K., Enderle D., Noerholm M., Breakefield X.O., Skog J.K. (2021). Exosome-based liquid biopsies in cancer: Opportunities and challenges. Ann. Oncol..

[108] Yakubovich E., Polischouk A., Evtushenko V. (2022). Principles and problems of exosome isolation from biological fluids. Biochem. Suppl. Ser. A Membr. Cell Biol..

[109] Malapelle U., Pisapia P., Addeo A., Arrieta O., Bellosillo B., Cardona A.F., Cristofanilli M., De Miguel-Perez D., Denninghoff V., Durán I. (2021). Liquid biopsy from research to clinical practice: Focus on non-small cell lung cancer. Expert Rev. Mol. Diagn..

[110] Ludwig N., Whiteside T.L., Reichert T.E. (2019). Challenges in exosome isolation and analysis in health and disease. Int. J. Mol. Sci..

[111] Martins T.S., Vaz M., Henriques A.G. (2023). A review on comparative studies addressing exosome isolation methods from body fluids. Anal. Bioanal. Chem..

